# Nitrosporeusine analogue ameliorates Chandipura virus induced inflammatory response in CNS via NFκb inactivation in microglia

**DOI:** 10.1371/journal.pntd.0006648

**Published:** 2018-07-12

**Authors:** Abhishek Kumar Verma, Trushnal S. Waghmare, Gorakhnath R. Jachak, Satish Chandra Philkhana, D. Srinivasa Reddy, Anirban Basu

**Affiliations:** 1 National Brain Research Centre, Manesar, Haryana, India; 2 National Institute of Virology, Pune, India; 3 CSIR-National Chemical Laboratory, Pune, India; Molecular Biology Unit (MBU), INDIA

## Abstract

Chandipura Virus (CHPV), a negative-stranded RNA virus belonging to the *Rhabdoviridae* family, has been previously reported to bring neuronal apoptosis by activating several factors leading to neurodegeneration. Following virus infection of the central nervous system, microglia, the ontogenetic and functional equivalents of macrophages in somatic tissues gets activated and starts secreting chemokines, thereby recruiting peripheral leukocytes into the brain parenchyma. In the present study, we have systemically examined the effect of CHPV on microglia and the activation of cellular signalling pathways leading to chemokine expression upon CHPV infection. Protein and mRNA expression profiles of chemokine genes revealed that CHPV infection strongly induces the expression of CXC chemokine ligand 10 (CXCL10) and CC chemokine ligand 5 (CCL5) in microglia. CHPV infection triggered the activation of signalling pathways mediated by mitogen-activated protein kinases, including p38, JNK 1 and 2, and nuclear factor κB (NF-kappaB). CHPV-induced expression of CXCL10 and CCL5 was achieved by the activation of p38 and NF-kappaB pathways. Considering the important role of inflammation in neurodegeneration, we have targeted NF-kappaB using a newly synthesised natural product nitrosporeusine analogue and showed incapability of microglial supernatant of inducing apoptosis in neurons after treatment.

## Author summary

Chandipura virus is a negatively stranded single RNA virus, which has claimed many lives in several outbreaks in India and Indian subcontinent. Our previous study shows CNS inflammation plays a vital role in inducing neurodegeneration post infection. In continuation to that our present study systematically examined the mechanism of microglial activation and function of NF-kappaB in CNS inflammation. We have shown that though CHPV infection is not productive in microglia, it leads to NF-kappaB activation that regulates secretion of cytokines and chemokines. The uncontrolled expression of inflammatory molecule disturbs the brain homeostasis leading to alteration of tight junction proteins that are an essential component in Blood-Brain Barrier. The expression of CCL5 and CXCL10 initiates peripheral leukocytes recruitment at the site of infection leading to aggravated inflammatory response, which has the deleterious effect on neurons. Our result shows nitrosporeusine targets microglial activation, and inhibits inflammation in in vitro as well as in vivo. The inhibition of inflammation has a protective effect on brain leading to enhance survival in mouse model.

## Introduction

Microglia, the developmental and functional equivalents of macrophages in somatic tissues [1], exert a central role in a host defence and immune surveillance against infectious agents in the central nervous system (CNS) [2]. Microglia being multitasking act as scavengers and antigen-presenting cells in the CNS, control the proliferation of astrocytes and produce soluble factors associated with an immunologic response [3],[4]. Under physiological conditions, microglia exist in a quiescent state lacking many of the effector functions and receptor expression patterns observed in macrophages within other tissues. However, in response to pathogen infection in the brain, microglia readily transform into an activated state, acquiring numerous if not all of the macrophage properties required to launch effective immune responses [5]. During viral infection activated microglia respond through a highly regulated network of cytokines and chemokines, which subsequently recruits the peripheral leukocytes into the CNS and orchestrate a multicellular immune response against the infectious agent [5].

Leukocytes are recruited into the CNS involves a sequence of process that can be mediated by chemokines. Chemokines are low molecular- weight and structurally related signalling molecules that are divided into four subfamilies, designated C, CC, CXC, and CX3C chemokine ligands based on the positions of their cysteine residues [6]. These molecules orchestrate efficient trafficking and recirculation of the leukocyte population within the blood vessels, lymph, lymphoid organs, and tissues, a necessary process during host immune surveillance and in acute and chronic inflammatory responses [7],[8]. Increasing evidence suggests that CNS-resident cells secrete various kinds of signalling chemokines upon injury or infection and that attracts peripheral leukocytes, such as lymphocytes, monocytes, transmigrate toward the chemokine gradient, breaching the blood-brain barrier, and gain access to the brain parenchyma [9],[6].

Most of the chemokines expression is regulated primarily at the level of transcription through activation of a definite set of transcription factors, such as nuclear factor κB (NF-kappaB) and interferon (IFN) regulatory factors [10]. It has also been reported that signal transduction pathways mediated by the mitogen-activated protein kinase (MAPK) family, including c-Jun N-terminal kinase (JNK), and p38, contribute to the activation of transcription factors [11],[12]. Environmental stresses, such as bacterial endotoxins, proinflammatory cytokines, osmotic shock, UV irradiation, and virus infections are reported to activate p38 and JNK [13],[14].

CHPV belonging to the Rhabdoviridae family has been a severe threat to the population in the Indian subcontinent, and several outbreak has claimed many lives for more than a decade [15]. It mainly infects children below 15 years and characterises influenza-like symptoms. It is a neurotropic virus that is transmitted majorly through sand flies, mosquito and ticks [16]. There is no specific treatment available to date; symptomatic treatment involves the use of mannitol to reduce brain edema. Previous studies, as well as data available from our lab, shows the role of microglia secreted inflammatory molecules in neurodegeneration in CHPV infected mouse [17],[18],[19].

In the present study, we have systemically examined the effect of a Nitrosporeusine derivative (-)-25b, an anti-inflammatory molecule against CHPV infection. Compound (-)-25b is one of the optimised compounds from the library of analogues synthesised based on a natural product Nitrosporeusine alkaloid of marine origin and has found to be effective against LPS induced inflammation in the mouse. Here, we have used Nitrosporeusine analogue (-)-25b, which was found to show the best activity against CHPV infection. Previously, we have demonstrated the capability of virus-induced microglial supernatant in inducing neuronal death in HT22 cells as well as primary neurons [17]. In this work we have targeted inflammation using nitrosporeusine analogue and showed the delayed survivability and disease progression post-CHPV infection. We also show that CHPV infection of microglia actively induces the gene expression and protein production of two chemokines, CXC chemokine ligand 10 (CXCL10) and CC chemokine ligand 5 (CCL5). The activation of NF-kappaB and chemokines upregulation leads to infiltration of peripheral leukocytes and monocytes further deteriorating the condition. Furthermore, our data indicate that the CHPV-induced production of CXCL10 and CCL5 is positively and negatively regulated by the activation of cellular signalling pathways mediated by and NF-kappaB. Nitrosporeusine analogue (-)-25b directly or indirectly targets NF-kappaB activation, further inhibiting sequence of disease leading to the improved disease condition.

## Materials & methods

### Ethics statement

All animal experiments were approved by the Institutional Animal and Ethics Committee of the National Brain Research Centre (approval no. NBRC/IAEC/2013/88). The animals were handled in strict accordance with good animal practice as defined by the Committee for Control and Supervision of Experiments on Animals, Ministry of Environment and Forestry (CPCSEA), Government of India.

### Virus and cell

CHPV (strain no. 1653514 isolated from the human patient in Nagpur, 2003) was propagated in Vero cell line. The virus was propagated in the Vero cell line, and viral titer was measured using plaque assay, which was found to be 3×10^9^ pfu/ml. HT22 (immortalised mouse hippocampal neuronal cell line was gifted by Dr Shiv Kumar Sharma, National Brain Research Centre) cells were used for our experiment with prior permission from Dr Dave Schubert of Salk Institute from whom these cells were initially obtained. HT22 cells were grown at 37 °C in Dulbecco’s modified Eagle medium (DMEM) supplemented with 3.7%, Sodium bicarbonate (Sigma, USA), 10% FBS (Gibco, Thermo Fisher Scientific, USA) and penicillin-streptomycin(Sigma, USA).

Mouse microglial cell line N9 was gifted from Prof. Maria Pedroso de Lima, Center for Neuroscience and Cell Biology, University of Coimbra, Portugal. The cell lines were grown at 37 °C in Roswell Park Memorial Institute medium (RPMI-1640) supplemented with 10% fetal bovine serum, 100 units/ml penicillin, and 100 μg/ml streptomycin.

### Nitrosporeusine treatment

Nitrosporeusines A and B are two recently isolated marine natural products with a novel skeleton and exceptional biological profile. Our previous data suggest it as a potential candidate possessing anti-inflammatory property [20], so we planned and screened racemic as well as enantiopure forms. Preparation of several analogues followed the natural product synthesis, and all the synthesised compounds were evaluated for in vitro and in vivo anti-inflammatory potential. After extensive screening, an enantiomer (-)-25b was found to be most effective in CHPV induced inflammation. Animals were injected with 20mg/Kg body weight intraperitoneally.

### Infection and treatment of cells

N9 microglial cells were used for experimental purpose. N9 cells were cultured in 10% serum containing media and were seeded in 60 mm plate at the density of 5×10^5^. After 12 hours the media was replaced by serum-free media to limit the growth rate of the cells so that a particular number of cells can be monitored for infection. Post 2 hours incubation N9 cells were infected with CHPV at the multiplicity of infection (MOI) 0.1 for 1 hours, and then cells were washed twice with 1X PBS to remove non-internalised virus present in media and then, nitrosporeusine (-)-25b was added at the concentration of 100μM. Cells at various post-infection time points were harvested, and the culture supernatants were collected and stored at − 80 °C.

### TUNEL assay

Mouse hippocampal cell line HT22 was plated at the density of 10^5^ cells/well in 2-well chamber slide with serum containing media. Cells were incubated with UV inactivated N9 supernatant as per infection paradigm mentioned previously. At 12 hours post-infection period, cells were subjected to *In situ* Cell Death Detection Kit, TMR red as per the manufacturers’ guidelines (Roche, Germany). Brain sections were washed with 1X PBXT and then blocked in 5%BSA for 1 hours. Further tissue was processed with TUNEL kit as described.

### Cell counting

An equal area from every section was taken, and cells were counted from it. Five sections were scored for each sample and average was calculated. Counting was done manually using ImageJ software.

### Immunoblotting

N9 cells and mouse brain tissues under different treatment conditions were harvested for obtaining total cellular extracts, and the protein isolation procedure and immunoblotting steps were performed according to standard procedure. After being blocked with 5% skimmed milk, the membranes were incubated with primary antibodies against NF-kappaB (Cell signalling, USA), Cleaved caspase-3 (Abcam, USA), iNOS (Abcam, USA), Cox-2 (Santa Cruz, USA), p-p38(Cell signalling, USA), p-JNK (Cell signalling, USA), p-Iκκα/β (Cell signalling, USA), p-Akt (Cell signalling, USA), p38 (Cell signalling,USA), JNK (cell signalling,USA), NF- kappaB (Cell signalling,USA), Akt (Cell signalling,USA), Claudin-1 and occludin (a kind gift from Dr. Guruprasad Medigeshi, Translational Health Science and Technology Institute, Faridabad), β-catenin (Abcam,USA. a gift from Dr. Ellora Sen, NBRC) and CHPV (a kind gift by Bharat Biotech International Limited, Hyderabad, India) at 1:1,000 dilutions. After extensive washes with 0.1% PBS-Tween, blots were incubated with the Anti-Rabbit peroxidase-conjugated secondary antibodies (Vector Laboratories, USA). The blots were then processed for development using chemiluminescence reagent (Millipore, USA). The images were captured and analysed using the Uvitech Cambridge using NineAlliance software (Uvitech, United Kingdom). β -actin antibody (Sigma, USA) at 1:10,000 dilutions was used as loading control.

### ROS measurement

Intracellular ROS production in Mock and treated cells was assessed using the cell permeable, non-polar H_2_O_2_ sensitive dye 5-(and-6)-chloromethyl-2′, 7′—dichloro dihydro fluorescein diacetate (CM-H2DCFDA) (Sigma Aldrich, USA) as described previously [17]. The extent to which H_2_O_2_ is generated is defined as the extent of ROS generation. Briefly, N9 cells of the different group, i.e. mock, CHPV infected and (-)-25b treated group were cultured, and then in serum-free media, it was infected and treated with (-)-25b. Incubation in serum-free media further followed this for 3 hours after which, the cells were further treated with H2DCFDA (1 μM) for 30 minutes at 37 °C. Cells were washed twice with 1 × PBS, and fluorescent intensity of the cells was measured using in BD FACS verse in FACSuit software.

### Plaque assay

Plaque assay was performed following previously published protocol [17]. Vero cells were grown till complete monolayer formation in 10% FBS containing DMEM seeded in 6 well plates at the density of 4×10^4^ cells/well. After complete monolayer formation was achieved serum-containing media was changed to serum-free media and incubated for 2 hours to acclimatise the cells for serum starvation. Meanwhile, serial dilution was prepared in serum-free DMEM starting with a 1:10 dilution of the stock solution. The stock solution was serially diluted using 10-fold dilutions. Each dilution was added to each well of Vero cells. After two hours of incubation with the respective dilutions at 37 °C, supernatants were removed and washed twice with 1X PBS to avoid multiple infection cycles. 3ml of agarose overlay (9ml 2% agarose (Roche, Germany), 10ml 2X Minimal essential media (Sigma, USA), 1ml FBS (Gibco, Thermo Fisher Scientific, USA), 100μL penicillin-streptomycin (Sigma, USA)) was then added to each well. The plate was kept at 4 °C for solidifying the overlay after which it was returned to 37°C for incubation of 24 hours. 4% PFA was added post-incubation period for fixation of the cells for further analysis. Subsequently, the overlay was removed, and cells were stained with crystal violet and plaque was counted. The viral titers were expressed as PFU/ml, calculated as [(number of plaques per well) × (dilution)]/(inoculum volume).

### RNA isolation and real-time PCR (qPCR)

Mouse brain tissue and harvested N9 cells were homogenised using trizol reagent as per manufacturers’ protocol (TRI reagent, Sigma, USA). For qPCR analyses, cDNA was synthesised using Advantage RT-for-PCR kit (Clontech Laboratories, CA). Oligonucleotide primers specific genes, e.g. CHPV, CCL5, CXCL10, I-CAM, VCAM and MMP-9, were used as mentioned in Table 1. Power SYBR Green PCR master mix (Applied Biosystems) was used for the experiment. The qPCR results were analysed as per the user manual guidelines.

**Table 1:** 

CHPV	Forward- 5’-ACC TGG CTC CAA ATC CAA TAC-3’
	Reverse- 5’-GGT GGa TCA GAC GGA GAG ATA-3’
ICAM	Forward- 5’-GTCCGCTGTGCTTTGAGAACT-3’
	Reverse- 5’-CGGAAACGAATACACGGTGAT-3’
VCAM	Forward- 5’-GGGACGATTCCGGCATTTAT-3’
	Reverse- 5’- TTCAACTGATTTTCTGCTAATT-3’
MMP-9	Forward- 5’-ATCTCTTCTAGAGACTGGGAAGGA-3’
	Reverse-5’-AGCTGATTGACTAAAGTAGCTGGA-3’
CCL-5	Forward-5’-TGCCCACGTCAAGGAGTATTTC-3’
	Reverse-5’-AACCCACTTCTTCTCTGGTTG-3’
CXCL-10	Forward- 5’-GCCGTCATTTTCTGCCTCAT-3’
	Reverse- 5’-GCTTCCCTATGGCCCTCATT-3’

### Cytokine bead assay

The CBA kit (BD Biosciences, NJ, USA) was used to quantitatively measure cytokine levels in the N9 cells supernatant as well as mouse brain lysate. 50 μl of mouse inflammation standard and sample dilutions were used to perform, the assay according to the manufacturers’ instructions and analysed on the BD FACS Verse (Becton Dickinson, CA, USA). A similar protocol was followed to analyse the cytokine levels for brain samples post-CHPV infection.

### Nitric oxide (NO) assay

Nitric oxide released from N9 supernatant following CHPV infection was assessed using Griess reagent as described previously. Briefly, 100 μL of Griess reagent (Sigma, St. Louis, USA) was added to 100 μL of supernatant and incubated in the dark for 15 minutes. The intensity of the colour developed was estimated at 540 nm with the help of a Benchmark plus 96-well ELISA plate reader (Biorad, CA, USA). The mean fluorescent intensity was plotted for each sample.[17].

### Immunohistochemistry

Fluorescence immunohistochemistry was performed for Mouse anti-Iba-1(1: 300, Chemicon, USA) for activated microglia. For peripheral immune cells CD3 (1:200, Millipore, USA,) a marker for activated T-cells, CD-68 (1:400, Abcam, USA) a monocyte lineage marker and CD11b (1:200, Millipore, USA) a macrophage marker were used in brain samples. The corresponding secondary antibodies were used: goat anti-mouse Alexa Fluor 488 (1:1000; Molecular Probes) for Iba-1, goat anti-mouse Alexa Fluor 594 for both CD3, CD11b and Rabbit anti-Rat fluorescein for CD68 Cell nuclei were stained with DAPI.

### ELISA

ELISA was performed to examine protein expression level of chemokines. N9 cells were cultured and seeded in 60 mm dish, and then standard infection was followed as described earlier. Then supernatants at the different time point were collected, and ELISA was performed as protocol described. In short, plate was coated with antibody of CXCL10 (R&D System, USA) and CCL5 (R&D System, USA) (diluted in coating buffer to 1 μg/ml) using coating buffer and then incubated for overnight. Next day after blocking for an hour in blocking buffer (1% BSA in PBS) supernatant was added to each well (100 μl) and incubated at RT for 6 hours, followed by three PBST wash. Then secondary biotin antibody (R&D System, USA) was added in each well (100 μl diluted in blocking solution 1 μg/ml), and incubated at RT for 30 minutes followed by three PBST wash and then incubation with streptavidin (Vector Laboratories) for 30 minutes at RT. The substrate (TMB solution, Vector Laboratories) was added and was incubated for 20 minutes and then stop solution was added for 100μl per well. Optical Density was measured at 450nm (Tecan infinite M200Pro, Switzerland).

For mouse brain samples ELISA was performed from mouse brain protein samples using protocol mentioned above.

### Inhibition of cellular signalling pathway

Inhibition of MAPK, Akt and NF-kappaB signalling in microglia was carried out by specific inhibitors. Briefly, cells were incubated for 1 hours at 37°C in test media containing SB202190 for p38, SP600125 for JNk, LY294002 for Akt and Dexamenthasone for NF- kappaB at 10μM concentration were used just before the experiment and were subjected to the analyses as mentioned above in the presence of these inhibitors. Under the assay conditions, these inhibitors did not induce any cytotoxic effects as judged by a dye exclusion test using trypan blue.

### Virus inactivation

CHPV inactivation was carried out with a UV cross-linker (UVC 500, Hoefer scientific, USA) using short-wavelength UV radiation (UVC, 254 nm) at a distance of 5 cm for 25 minutes on ice as described earlier [17]. Inactivation of virus was verified by plaque assay for all three sets of treated supernatant which showed the absence of viral plaque formation in the UV treated culture supernatant.

### Statistical analysis

Experiments with paired treatment were analysed by t-tests. Experiments with >2 treatments were analysed by ordinary one-way analysis of variance (ANOVA) as appropriate with Holm-sidak correction for multiple tests. Prior to analysis data were tested for adherence to normality using the Shapiro-Wilk normality test. All analyses were conducted using GraphPad 13.0.

## Result

### Nitrosporeusine analogue reduces the severity of the infection

**Fig 1:**
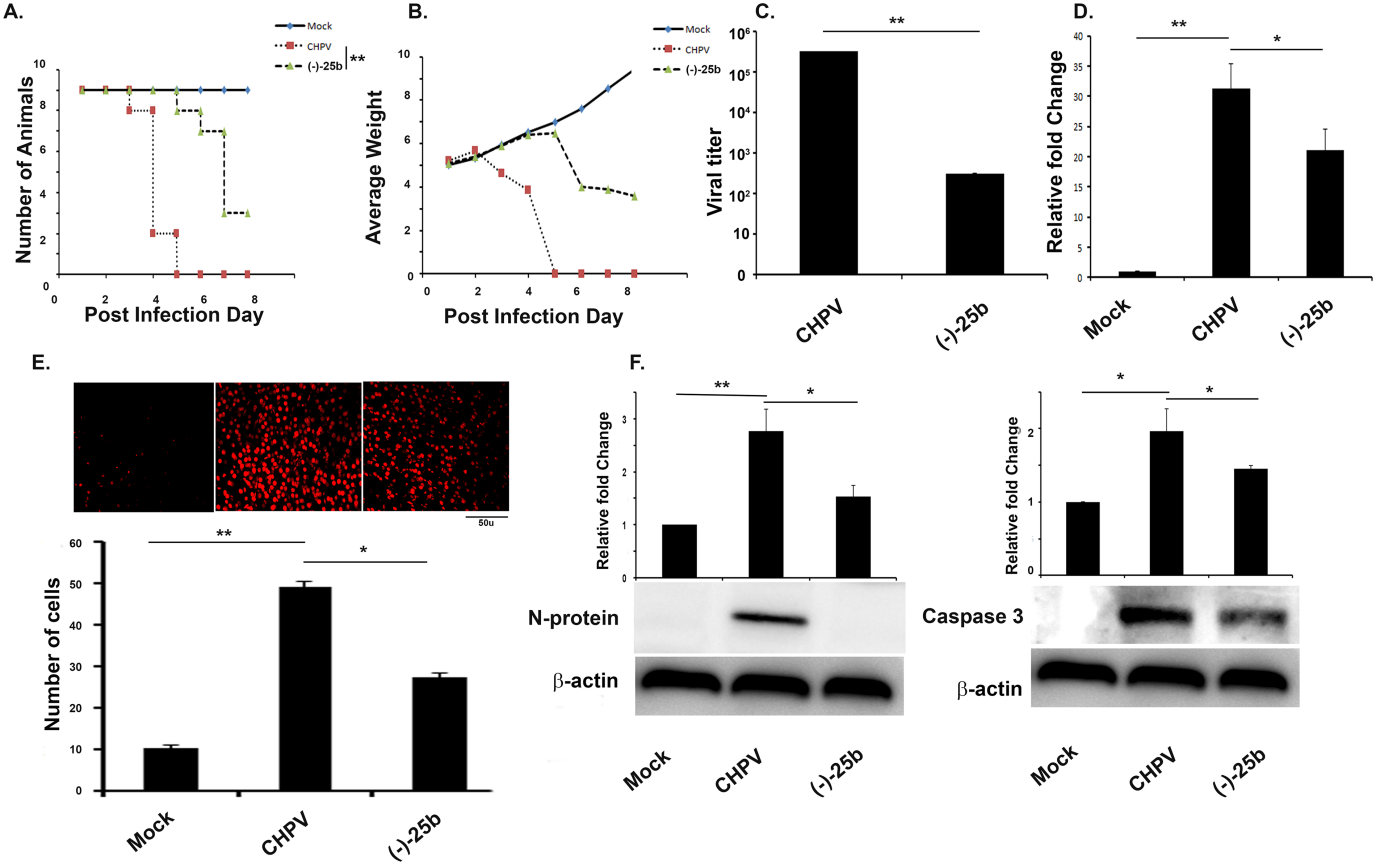
Nitrosporeusine treatment reduced the severity of infection and increases survivability in BALB/c mouse. A) Kaplan-Meier plot showed an increase in survivability of animals after (-)-25b treatment by 48–72 hours (2–3 days). B) Growth plot showed a comparatively delayed decrease in weight of (-)-25b treated group against only CHPV infected group. C) Plaque assay was performed on Vero cells inoculated with brain homogenate obtained from CHPV infected and CHPV infected-(-)-25b treated groups. Significant low number of plaques was observed in Vero cell culture infected with (-)-25b treated group when compared to the untreated group. D) qPCR analysis showed a significant reduction in CHPV mRNA level after (-)-25b treatment when compared to only CHPV infected group. E) TUNEL assay showed a decrease in TUNEL positive cells after minocycline treatment in CHPV infected, and (-)-25b treated brain sections. Bar graph was plotted denoting number of TUNEL positive cells in different samples. F) Western Blot for Viral M-protein and cleaved Caspase 3 showed significant decrease in expression after minocycline treatment in CHPV infected brain samples, which was quantified by densitometry graph. Validation of results were done by 3 independent experiments with at least 4 animals in each group.*p < 0.05, **p < 0.01.

Three different groups were mock treated, CHPV infected and nitrosporeusine treated after infection were analysed for survivability after infection. Kaplein-Meir graph plotted for our data shows mice treated with compound (-)-25b has enhanced survivability as compared to CHPV infected group (Fig 1A) Drastic weight loss is inevitable post infection, so we checked for disease progression by recording weight loss of animal post infection and we found delayed onset of symptom in compound (-)-25b treated group (Fig 1B). After the appearance of full symptom in CHPV infected mice brain sample was collected from all three groups for experiments. Next was we analysed viral load in the brain samples through plaque assay and found a significant reduction in viral load in analogue treated samples (Fig 1C). Similarly, qPCR data for the viral gene was found to be consistent with previous data (Fig 1D). Moving on next, we checked for cell death using TUNEL assay as neurodegeneration is the hallmark of CHPV infection [21]. TUNEL data shows decreased positive cells in compound (-)-25b treated group as compared to only CHPV infected group (Fig 1E). Western blots of N-protein of CHPV and cleaved Caspase 3 showed the significant reduction in expression in nitrosporeusine treated samples (Fig 1F). Concomitantly these observations helped us to conclude that nitrosporeusine analogue hinders the replication of the virus in neurons and hence neurodegeneration in vivo.

### Nitrosporeusine analogue reduces microglial activation and inflammation

**Fig 2:**
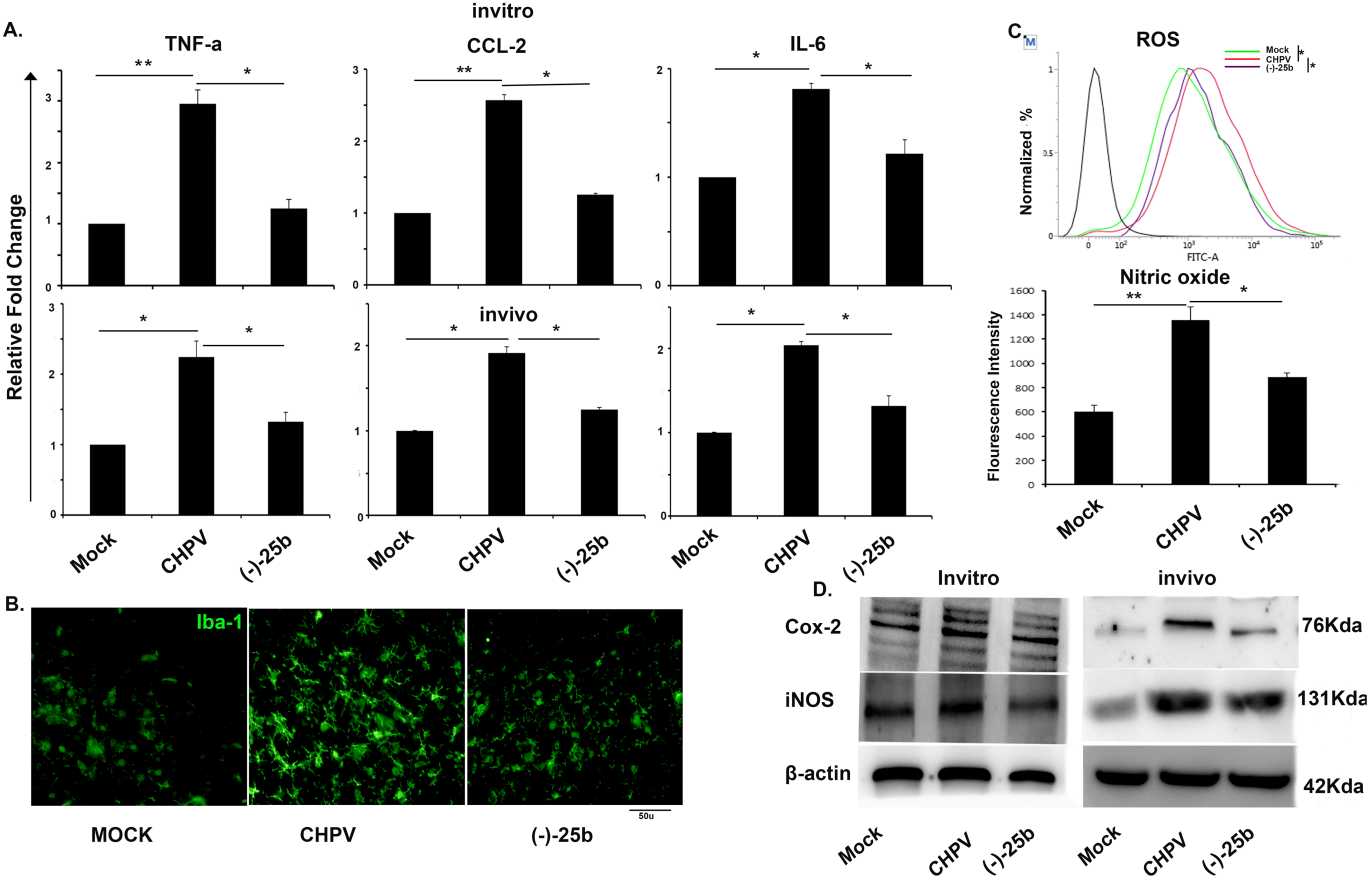
Nitrosporeusine inhibits CHPV induced microglia activation and reduces inflammatory molecules. A). CBA analysis was done from N9 supernatant and mouse brain protein to analyse expression level of cytokines. TNF-a, CCL2 and IL-6 shows enhanced expression in supernatant (6hpi) of N9. Our data suggest treatment of cells with (-)-25b compound is effective in inhibiting microglia mediated inflammation. Similarly, *in vivo* validation was performed in mouse brain (2dpi) and data was concomitant with in vitro result. B). Immunofluorescence staining for Iba-1 shows activated morphology of microglia. The star shaped morphology was more prominent in CHPV infected samples. The (-)-25b treated group shows decrease in activated morphology of microglia. C). ROS level using DCFDA shows decrease level in (-)-25b treated samples as compared to CHPV treated samples. NO generation was measured from N9 cells using Griess reagent. NO was measured from N9 supernatant shows increase level of NO in CHPV infected samples as compared to (-)-25b treated group at 6 hpi. D). Western blot analysis of Cox-2 and iNOS shows decrease expression level in (-)-25b treated group in in vitro as well as in vivo samples which was found to be upregulated in CHPV infected group.

Chronic activation of microglia leads to secretion of cytokines that have the deleterious effect on neurons and acts as a significant player of neurodegeneration post-CHPV infection [21]. We investigated the functional profile of activated microglia by CBA analysis of inflammatory cytokines in N9 cells at 6 hpi revealed the effectivity of (-)-25b against the inflammation induced by CHPV infection. Analysis of TNF-α, CCL2 and IL-6 shows many-fold increase in cytokine level which decreases sharply in (-)-25b treated group as compared to only infected group (Fig 2A). The bar graph shows relative fold change in expression of cytokines with mock. We validated the same data in the animal brain at 2dpi and were found to be correlated with in vitro data. CBA analysis of TNF-a, IL-6 and CCL2 shows a sharp decrease in cytokine level post (-)-25b treatment. Bar graphs show relative fold change with mock (Fig 2A). Activated microglia are known for secretion of inflammatory mediators. Microglial activation was examined using Iba-1 antibody and found a decrease in the number of star-shaped cells, i.e. activated microglia in (-)-25b treated sample as compared to only infected samples (Fig 2B).

Generation of reactive oxygen species (ROS) and nitric oxide (NO) by microglial cells in response to infection is a crucial marker for oxidative stress in these cells. Further, we checked for ROS generation post CHPV infection which shows a decrease in ROS generation in (-)-25b treated samples. Nitric oxide measurement also indicates the reduction in its expression level in drug-treated samples (Fig 2C).

Our earlier reports suggest upon activation; microglia secretes iNOS and COX-2 [21]. We found a decrease in expression level of iNOS and COX-2 in N9 cells as well as brain samples (Fig 2D).

This data shows a significant reduction in inflammation upon (-)-25b treatment in virus-infected cells as well as in the brain.

### CHPV infection stimulates chemokine gene expression in microglia

**Fig 3:**
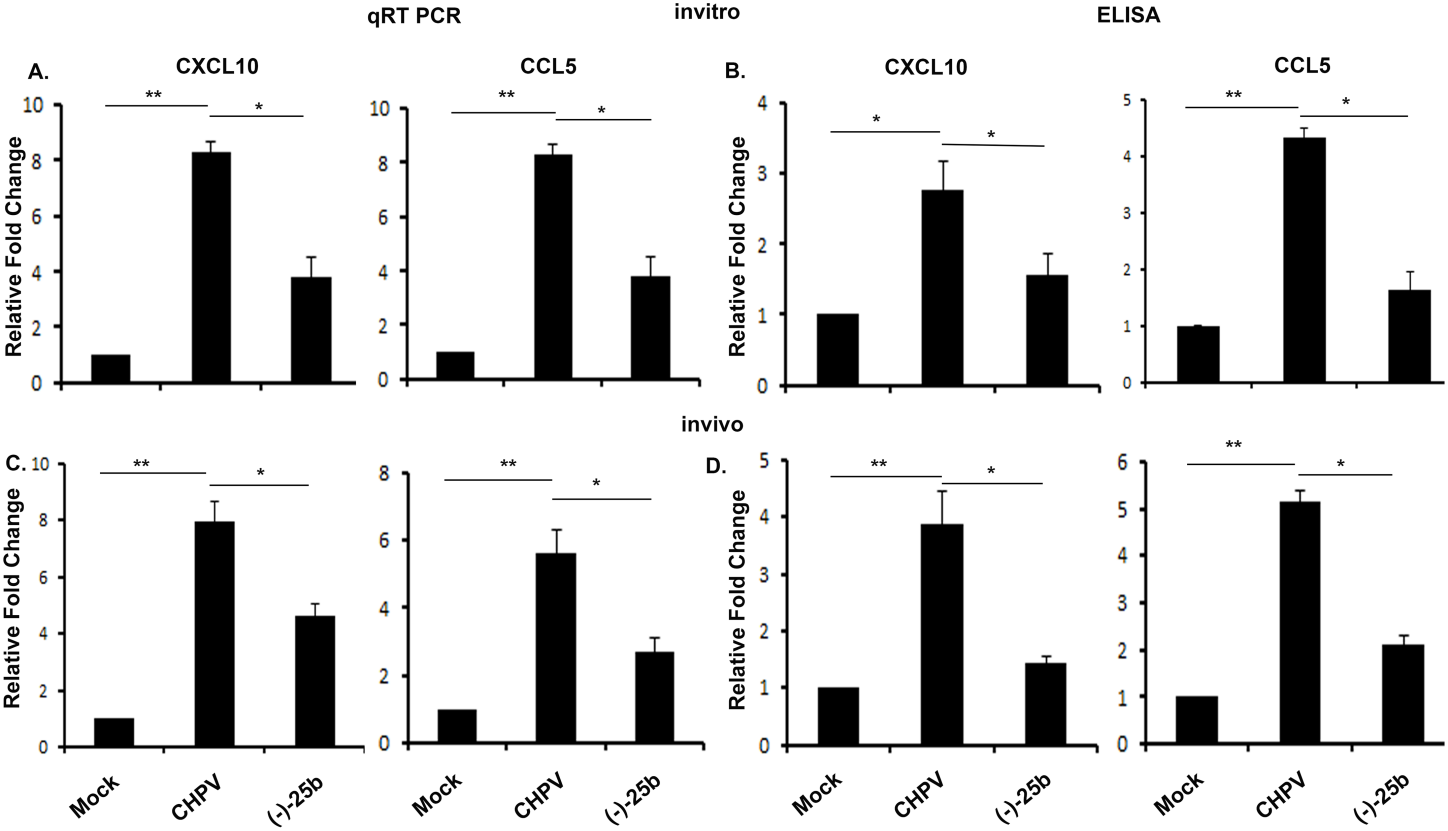
Chemokine expression increases post CHPV infection. A). mRNA expression level was checked for CXCL10 and CCL5 from N9 cell sample. The data shows significant increase in expression level of these chemokines in CHPV infected microglia which was found to be similar with control in (-)-25b treated group. B). Further to confirm the protein expression level, ELISA was performed. ELISA data shows upregulation in expression level in CHPV infected microglia. C &D). Chemokine expression was further validated in brain sample and was found to be consistent with N9 data showing increase in CHPV infected sample and decrease in (-)-25b treated sample. Experiments were performed at thrice before reaching to conclusion. In vivo experiment were performed with at least four animal in each group. *p < 0.05, **p < 0.01.

It is a well-documented fact that aggravated inflammation during viral infection leads to secretion of chemokines. These chemokines are meant for attracting immune cells at the site of infection, but uncontrolled increase creates a hostile environment for cells. We checked for different chemokines using qPCR and found a rise in CXCL10 and CCL5 post-CHPV infection (Fig 3A) in N9 cells. To check if enhance mRNA expression pattern is correlated with protein; ELISA was performed for CXCL10 and CCL5 proteins (Fig 3B). Our ELISA data for CXCL10 and CCL5 shows an increase in expression after CHPV infection in N9 cells at 6hpi, which decreases significantly post (-)-25b treatment.

Chemokine expression level was then checked in mouse brain sample. Our qPCR data for mRNA expression level shows a significant increase in CXCL10 and CCL5 at 2dpi. Then this data was further validated at protein expression level by ELISA. Our data was found to be correlated with PCR data suggesting enhanced expression of CXCL10 and CCL5 and were found to be a multifold increase in expression level of these chemokines at 2dpi (Fig 3C & 3D).

Taken together, these data demonstrate that the CHPV-induced expression of CXCL10 and CCL5 is triggered at the stage after viral infection.

### CHPV infection increases leukocytes infiltration and BBB permeability

**Fig 4:**
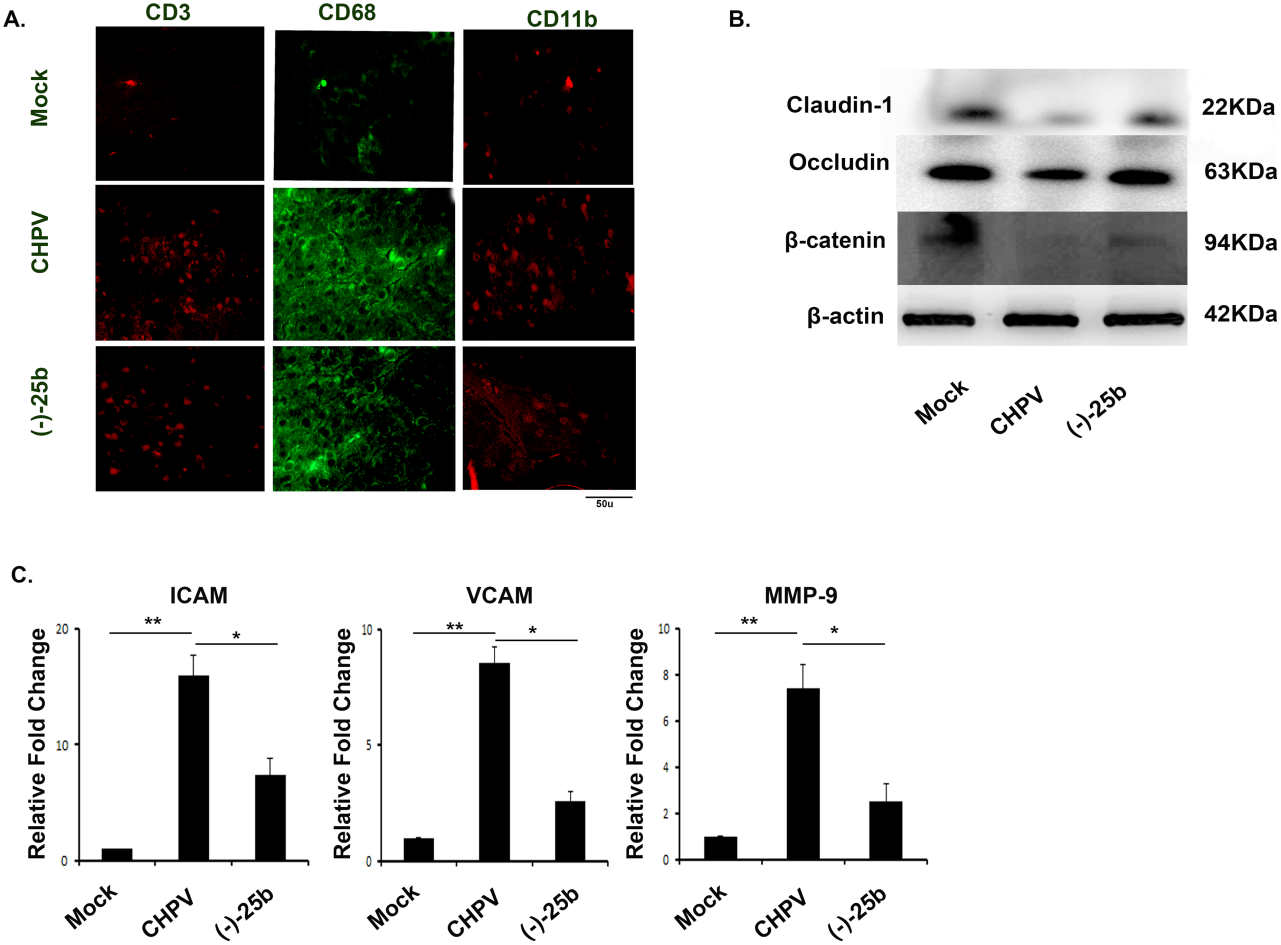
Leukocyte infiltration and BBB breaching during CHPV infection. A). Peripheral immune cells were stained to check their presence in brain post infection. CD3 positive cell were more prominent in CHPV infected samples. Similarly CD68 and CD11b positive cells were present in CHPV infected sample suggesting infiltration of peripheral cells in brain. B). To examine BBB breaching, we checked expression level of tight junction protein. Claudin-1, occludin and β-catenine was checked for western blot. Data suggest increase in expression of these proteins in CHPV infected samples. (-)-25b treated brain shows significant decrease in expression level of the proteins. C). mRNA expression was checked to analyse the status of ICAM, VCAM and MMP-9. Validation of results were done by 3 independent experiments with at least 4 animals in each group.*p < 0.05, **p < 0.01.

Enhanced chemokine level leads to infiltration of peripheral leukocytes and monocytes into the brain. To assessed the functional relevance of increased chemokines with leukocyte infiltration we performed IHC staining of brain sections which reveals the presence of CD3 positive cells in brain suggesting presence of activated T-cells which decreases significantly in (-)-25b treated section (Fig 4A). Similarly CD68 a monocyte marker and CD11b a macrophage lineage marker shows strong presence in infected brain samples (Fig 4A). Presence of peripheral cells encouraged us to check for BBB permeability. Occludin, Claudin -1 and β-catenin are tight junction protein which gets manipulated during pathogenic infection and blood-brain barrier breaching. We performed western blot to check expression level of these proteins in the presence of virus as well as in drug treatment. Our data show a significant decrease in expression of occludin post-CHPV infection which was found to be significantly higher in (-)-25b treated samples (Fig 4B). Similarly, claudin-1 and β-catenin shows similar data suggesting suppression of CNS inflammation control BBB breaching. We performed qPCR to check for the expression level of BBB regulatory genes. Our data shows enhanced expression of matrix metalloproteinases 9 (MMP-9), ICAM, & VCAM in CHPV infected brain which decreases sharply after (-)-25b treatment (Fig 4C).

Our data signifies BBB breaching and infiltration of peripheral cells in the brain after CHPV infection.

### CHPV induced MAPKs activation regulates chemokine expression

**Fig 5:**
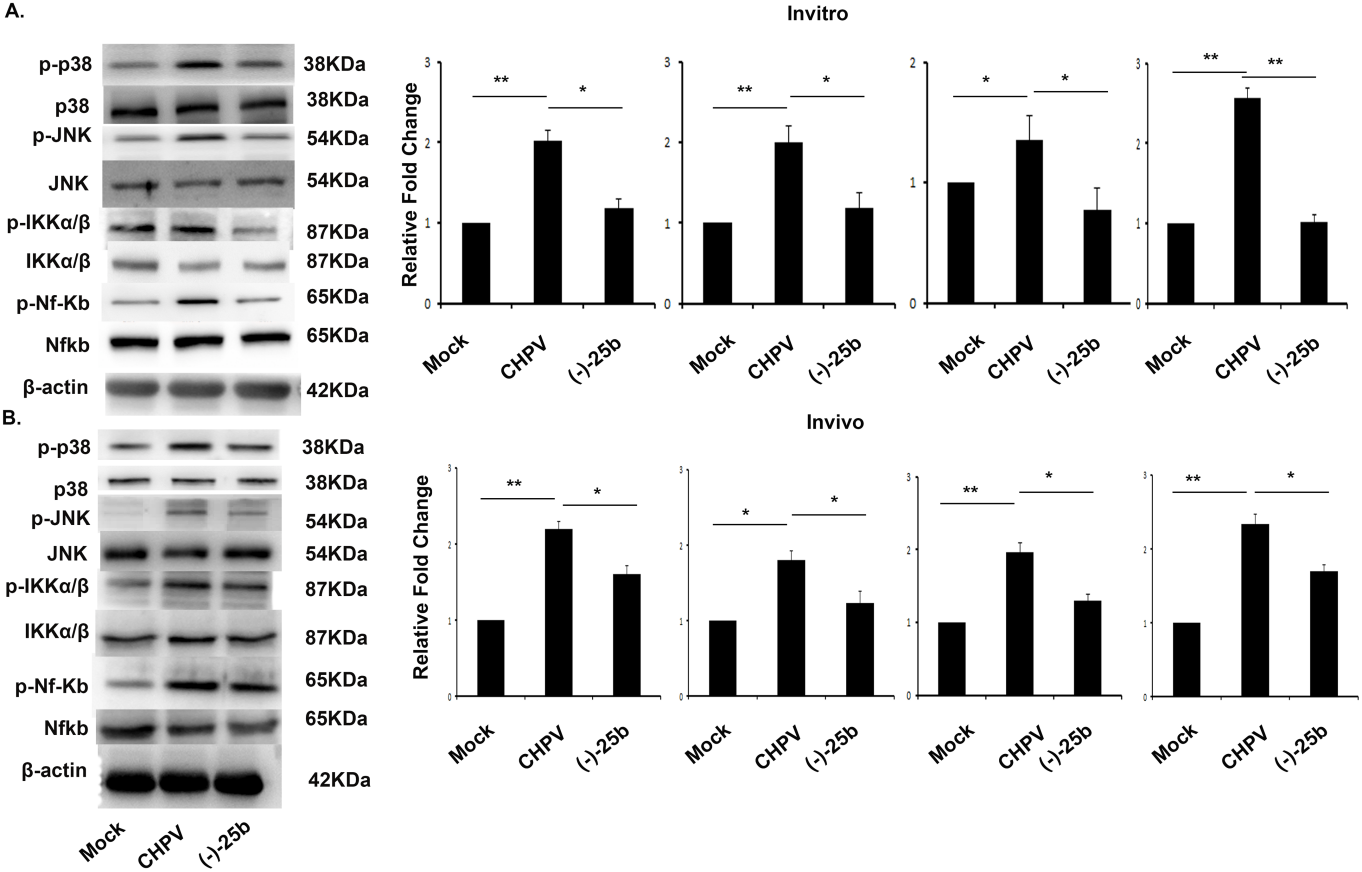
CHPV infection activates MAPK signaling pathways in microglia. (A) MAPK activation in microglia following CHPV infection. Cells were mock infected or infected with CHPV or (-)-25b treated followed by CHPV infection were processed for western blot. The protein levels of phosphorylated MAPKs and NF-kappaB were analyzed by Western blotting as described in the text. The amounts of β-actin were also assessed to monitor the equal loadings of protein extracts. Data shows upregulation in expression of p-p38, p-JNK, p-Iκκb, p-NF-kappaB proteins. (B) Further brain lysate were processed to check expression of these proteins. Our data shows increase in p-p38, p-JNK, p-Iκκa/b, p-NF-kappaB in CHPV infected group which decreases in (-)-25b treated samples. Experiments were performed at thrice before reaching to conclusion. In vivo experiment were performed with atleast four animal in each group. *p < 0.05, **p < 0.01.

A definite set of transcription factor governs chemokine expression. The enhanced production of CXCL10 and CCL5 in CHPV-infected microglia implies the possibility that CHPV infection may stimulate the cellular signalling pathway underlying chemokine expression. Considering the importance of MAPKs in gene regulation and NF-kappaB in disease and pathogenesis we examined activation level of NF-kappaB, p38, and JNK. In order to assess the activation of MAPK signalling pathways in microglia during the course of CHPV infection, cells were mock infected or infected with CHPV, and Western blotting examined the degrees of phosphorylation. We found CHPV infection in microglia leads to activation of NF-kappaB, p38, JNK, and IKKα/β in N9 cells. Western Blot analysis shows increase expression of NF-kappaB, p38, IKKα/β and JNK in CHPV infected cells which decreases in (-)-25b treated N9 samples at the early time point as 3hpi (Fig 5A).

We then moved to further validate the expression in brain samples. Mouse brain was processed for western blot, to check expression level of same genes as was in N9 cells. During early time of infection, brain protein shows increased expression of NF-kappaB, p38, IKKα/β and JNK in CHPV infected brain samples. Here also we found effectivity of (-)-25b and can significantly downregulate expression level of these proteins (Fig 5B). The bar graph shows relative fold change in expression level of proteins.

### NF-kappaB inhibitor is effective against CHPV mediated inflammation

**Fig 6:**
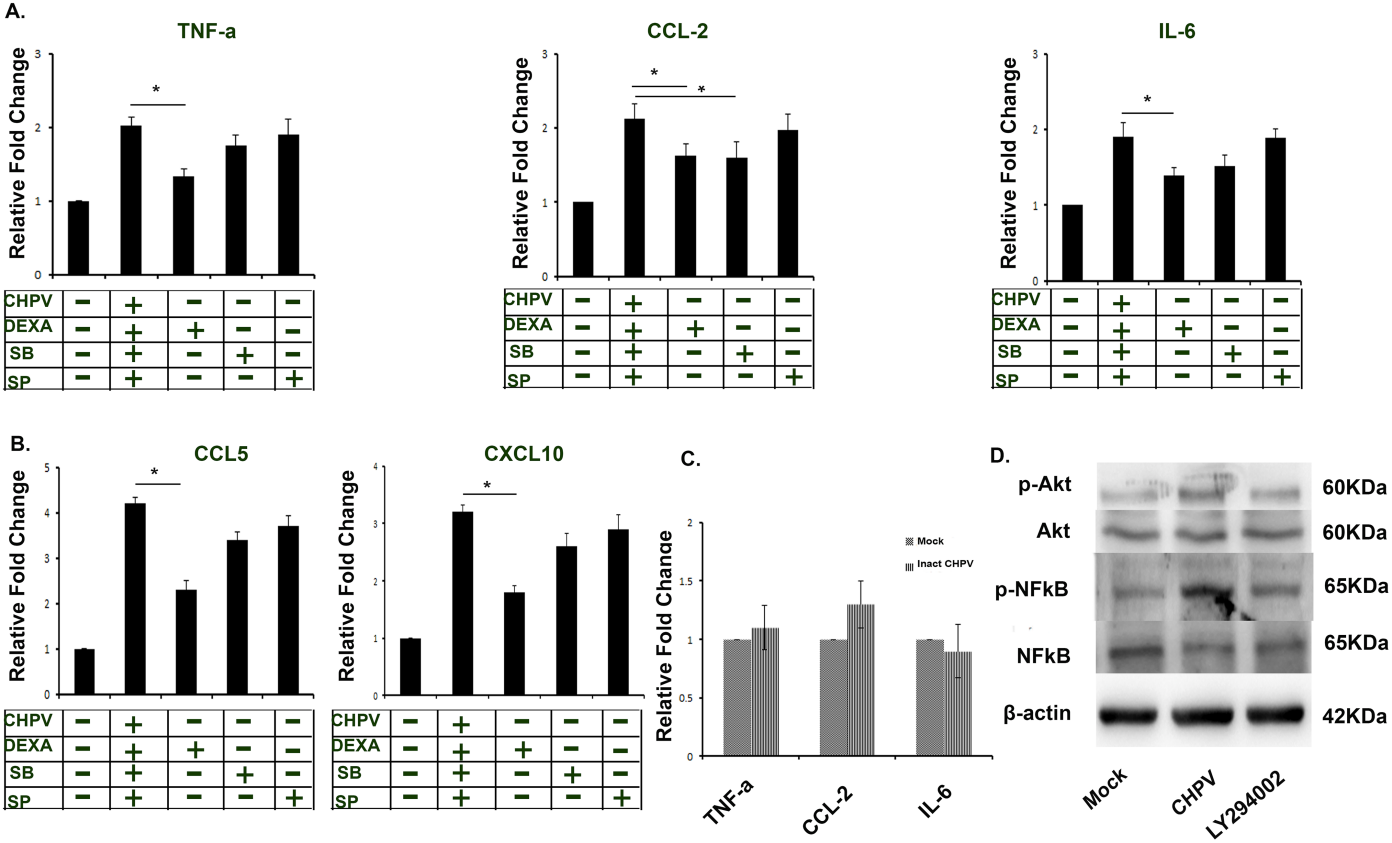
CHPV induces microglial activation through NF-kappaB pathway. A). p38 (SB239063), JNK (SP600125), Dexamethasone (Dexa) an inhibitor of NF-kappaB was used at concentration of 10μm to check their effect on CHPV induced cytokines and chemokines production. CBA analysis of cytokines expression shows decrease in expression level in Dexa treated inhibitors whereas other inhibitors were not very effective. Though SB239063 showed significance decrease in CCL2, but was not effective in downregulating TNF-α and IL-6. B). Chemokine expression level was checked in presence of inhibitors. Dexa was found to be most effective in inhibiting chemokine expression. C) CBA was performed in presence of inactivated virus to asses if live virus is required for virus dependent inflammation. CBA data shows incapability of inactivated virus in inducing cytokine expression. D). Western blot experiment shows role of Akt phosphorylation in activation of NF-kappaB. The data shows treatment of PI3K inhibitor (LY294002) at 10μM inhibited p-Akt level and thus NF-kappaB was inhibited.

Analysis of the CHPV-induced expression of cytokines and chemokines in the presence of MAPK inhibitors have revealed that the p38 and JNK pathways are not dominant in the production of these chemokines, but NF-kappaB inhibitor severely reduces cytokines level. We used the specific inhibitor of p38 (SB239063), JNK (SP600125) and NF-kappaB at a concentration of 10μM to check chemokines and cytokines production. Our data suggest dexamethasone; a known NF-kappaB inhibitor is potent in blocking cytokines and chemokines level as compared to other two inhibitors. The p38 inhibitor was found to be effective against the production of CCL-2 only whereas JNK inhibitor was not impressive against cytokines and chemokines generation (Fig 6A). Similarly, chemokine level was also found to decrease significantly in the dexamethasone-treated sample as compared to other inhibitors. This data suggest a critical role of NF-kappaB in CHPV mediated inflammation (Fig 6A & 6B). Now we wanted to explore if viral infection is required for the microglial based inflammatory response or just virus interaction is sufficient to induce activation. We used UV inactivated virus and infected N9 cells, and followed by analysis of cytokines level using CBA. Our data reveal inactivated viruses are incapable of inducing cytokine level. We didn’t find any significant change in the level of TNF-a, CCL-2 and IL-6 in inactivated virus treated sample as compared to mock (Fig 6C). This data suggest active virus is required for microglial activation.

The next question asked was the pathway through which NF-kappaB gets activated. Several reports, shows the importance of Akt in NF-kappaB activation and Akt as a downstream molecule in NF-kappaB activation [22]. Additionally it is also reported that PI3K/Akt pathway is involved in NF-kappaB dependent CCL5 secretion. We have demonstrated the role of p-Akt in activation of the NF-kappaB pathway in N9 cells. Using PI3K inhibitor (LY294002) at 10μM, which further inhibits Akt phosphorylation shows a decrease in NF-kappaB level (Fig 6D). This data shows inhibition PI3k/Akt signalling inhibits NF-kappaB activation, suggesting role of Akt pathway in CHPV induced NF-kappaB activation.

Our data confirmed the role of NF-kappaB activation in CHPV induced microglial activation that is taking place through PI3K/Akt pathway.

### Nitrosporeusine analogue inhibits microglial supernatant mediated death

**Fig 7:**
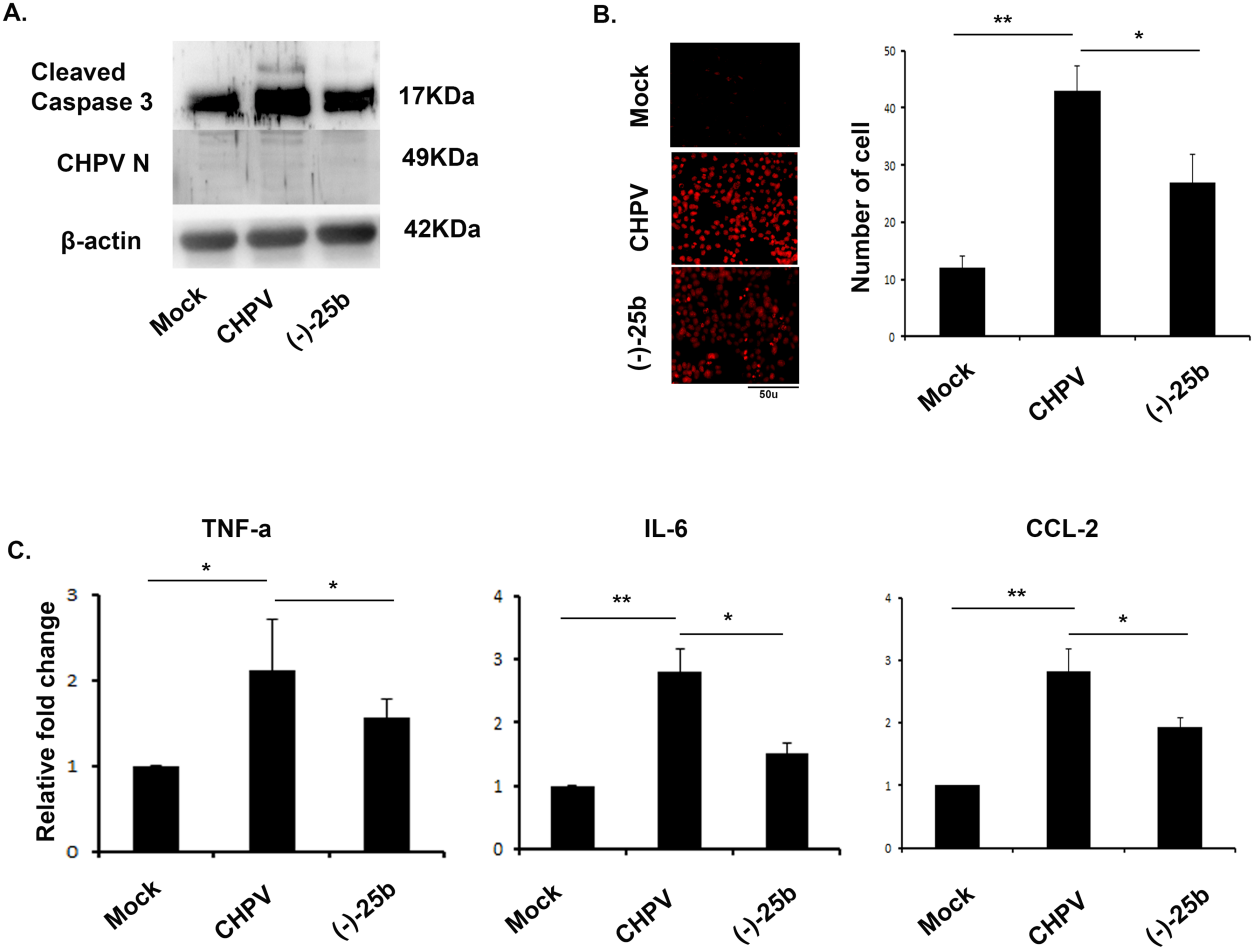
Bystander killing of neurons using supernatants from activated microglial cells. (**A**) Immunoblot image shows expression of caspase 3 in supernatant treated HT-22 cells. CHPV protein was checked to confirm complete absence of live virus particle in cells β -actin was used as loading control. (**B**) *In vitro* culture of HT-22 neuron showed TUNEL positive cells in supernatant treated panel confirming bystander killing of neurons. Bar graph shows number of positive cells in each sample. (**C**) CBA data revels presence of cytokines after UV-inactivation of supernatant suggesting role of cytokines in neuronal death.

CHPV activated microglial supernatant are capable of inducing neuronal death[21]. Here, we treated N9 cells with virus and in another group with virus and (-)-25b and then collected supernatant at 12 hpi. This supernatant was further UV-inactivated to get rid of any active viral particle. Our data show a significant reduction in caspase 3 level in (-)-25 treated cell supernatant as compared to the only virus infected supernatant in HT22 cells (Fig 7A). Virus protein load was checked to make sure absence of viral replication in HT22 cells to confirm the death is not because of virus (Fig 7A). TUNEL assay was performed to check cell death and was found to be correlated with western blot data. The UV treated supernatants were further analysed for CBA to confirm the presence of cytokines. This data shows nitrosporeusine treatment in N9 cells inhibits bystander death.

## Discussion

Microglia are resident immune effector cells within the CNS and are hence likely to get activated for encounter against infectious agents at very early stages of infection, as well as at later stages, when infiltration of peripheral leukocytes, such as lymphocytes and monocytes, are recruited into the brain parenchyma [23],[24]. Recruitment of leukocytes into the CNS is usually preceded by chemokine production from microglia and other CNS-resident cells, which is the first line of defence against infectious pathogens[25],[9]. CHPV replication is mainly limited to the neurons, and active replication has not been observed in any other resident cells of CNS [26]. However, in vitro studies provide evidence for the onset of viral gene expression in glial cells, implying that although CHPV virions were taken up by glial cells, in which virus gene expression occurs, the production of virus progenies is impaired at the later stage of the viral replicative cycle.

In response to enhanced survivability and delayed disease progression post-CHPV infection, we have systemically examined the cellular response of microglia to CHPV infection, and the activation of cellular signalling pathways leading to an aggravated inflammatory response in these cell types. Pro-inflammatory cytokines and chemokines released by the microglial activation process bind to specific receptors of neurons that initiate the apoptotic mechanism in the cells. In order to validate the effect of nitrosporeusine through inhibition of inflammation, we analysed the cytokine and chemokine levels from the N9 cells as well as brain samples (Fig 2). Following CHPV infection expression levels of TNF-a, CCL2 and IL-6 were found to increase that was previously implicated in playing decisive roles in encephalitis[27],[28] has decreased significantly after (-)-25b treatment. Regarding the cellular response of microglia to CHPV infection, we have observed expression of two chemokines, CXCL10 and CCL5, is notably induced in CHPV-infected microglia in N9 cells as well as in brain samples. Furthermore, the data obtained here exhibit that CHPV infection initiates activation of multiple signalling pathways mediated by NF-kappaB, p38, and JNK, in microglia and that viral gene expression is required for the activation of these signal-transducing molecules. Our data demonstrate NF-kappaB-dependent signal transduction is a critical process leading to the strong induction of CXCL10 and CCL5 expression in CHPV-infected microglia, and this signalling is indirectly augmented via the activation of the p38-mediated pathway. Our data also indicate that JNK, doesn’t contribute to the induction of CXCL10 expression and CCL5 expression as the inhibitor of JNK doesn’t reduce the expression level of chemokines (Fig 6B). The previous report demonstrated that the gene expression levels of chemokines, including CXCL10 and CCL5, in the CNS infected by Rabies virus are induced in the mononuclear cell [29]. Considering the strong chemotactic effects of CXCL10 and CCL5 on leukocytes, such as T cells and monocytes [30],[31], it is likely that the production of these chemokines is associated with the BBB breaching and infiltration of mononuclear cells into the CHPV-infected CNS. Though inflammation occurs, little information is available concerning the signalling of microglial activation and cascade of event occurs after CHPV infection in CNS. Our findings in the current study demonstrate the possibility that CNS-resident cells can produce CXCL10 and CCL5 via the recognition of CHPV infection. The data obtained here provide evidence for the CHPV-induced activation of intracellular signalling pathways in microglia. Recent extensive studies have indicated that microglia intrinsically produce CXCL10 and CCL5 upon infection with a variety of neurotropic viruses, including cytomegalovirus [32], human immunodeficiency virus[33], herpes simplex virus [34] Theiler’s murine encephalomyelitis virus [35], and Japanese encephalitis virus [36],[37]. However, the precise role of cell signalling molecules, especially that of MAPK subfamilies, in the CHPV-induced expression of these chemokines in microglia has not been studied before. As for NF-kappaB signalling being responsible for CXCL10 expression, a recent report suggests that the induction of CXCL10 production adenovirus infection is mediated by the Akt activation pathway[38]. The results obtained in the present study indicate that Akt phosphorylation is required for activation of NF-kappaB activation for CXCL10 expression in CHPV-infected microglia. Our findings are unique in that the activation of these MAPK pathways leading to CXCL10 and CCL5 expression is triggered at the step after virus entry because UV-inactivated CHPV virions failed to induce MAPK phosphorylation. It has been reported that p38, as well as PI3K/Akt is required for CCL5 production in astrocytes following infection with HIV infection [39]. Our data suggest inhibition of NF-kappaB leads to inhibition of CCL5 expression and Akt phosphorylation is required for NF-kappaB activation suggesting the role of this axis in CCL5 and CXCL10 expression.

Leukocyte infiltration is preceded by chemokines expression. Leukocyte influx into the brain is a defining feature of viral encephalitis. It has long been assumed that leukocyte infiltration into the CNS is critical for virus clearance and recovery[40]. Alternatively, infiltrating leukocytes may paradoxically contribute to a more severe outcome that results from the destruction of neuronal cells. Chemokines are pivotal regulators of leukocyte trafficking [41],[6]. In the context of CHPV infection, it is not clear the mechanism for induction of inflammation and chemokines are essential for the attraction of leukocytes into the CNS and what role they play during viral infection of the brain, or indeed what impact this has on encephalitis-associated morbidity and mortality.

Our data shows CNS leukocyte recruitment, and aggravated inflammatory response modulate host immune responses, which in excess may otherwise contribute to virus-induced damage and mortality. Nitrosporeusine is newly synthesised drug acts as an anti-inflammatory agent [20]. In our case, we have found that nitrosporeusine treatment in animal significantly increases animal survivability and decreases disease progression in BALB/c mouse (Fig 1A & 1B). The decrease in viral load, as well as caspase level, intrigued us to investigate the mechanism of action of this drug. Inflammatory mediators are known to induce neurodegeneration if produce uncontrollably [19]. NF-kappaB is known to play a very crucial role in inflammation and also reported to get activated during viral infection [42],.[43].

In conclusion, our work defines the mechanism of CHPV induced microglia-mediated death in CNS. We have shown the activation of NF-kappaB in microglial cells post CHPV infection. This activation is required for secretion of deleterious chemokines and cytokine that plays a pivotal role in recruiting peripheral immune cells at the CNS, thus further aggravating the condition. We have shown the therapeutic role of nitrosporeusine analogues, in particular, enantiomer (-)-25b in CHPV infection. Our data suggest compound (-)-25b inhibits microglial NF-kappaB activation and hence inhibits chronic inflammation in the brain.
